# 148. Host-Response Testing to Guide Antibiotic Prescription: Association Between MeMed BV® Results and Clinical Outcomes in an Urgent Care Network

**DOI:** 10.1093/ofid/ofaf695.050

**Published:** 2026-01-11

**Authors:** Boaz Kalmovich, Daniella Rahamim_Cohen, Shirley Shapiro Ben David

**Affiliations:** Maccabi Healthcare Services, Tel Aviv, Tel Aviv, Israel; Maccabi Healthcare Services, Tel Aviv, Tel Aviv, Israel; Maccabi Healthcare Services, Tel Aviv, Tel Aviv, Israel

## Abstract

**Background:**

Inappropriate use of antibiotics is particularly prevalent in urgent care centers (UCCs), where diagnostic uncertainty is higher due to limited time and diagnostic tests. MeMed BV® (MMBV), an FDA-cleared host-response test that aids in distinguishing bacterial from viral infections by integrating levels of three immune proteins into a bacterial likelihood score, has NPV >98%.

We evaluated the relationship between concordance of antibiotic prescription at the UCC with MMBV results and patient outcomes in post-UCC 7-day follow-up.

**Table 1:**
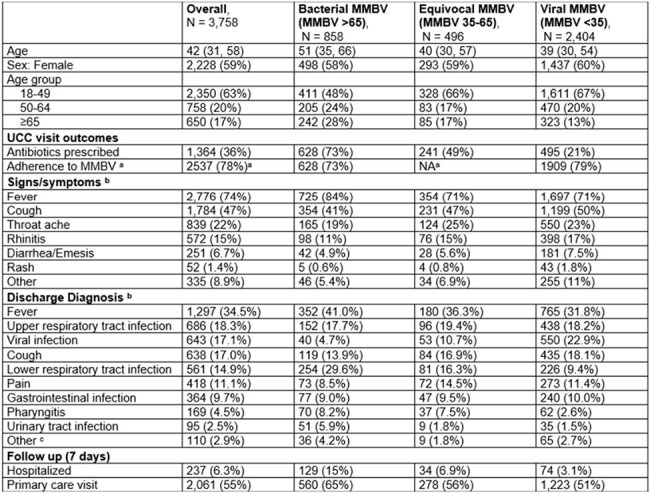
Patient characteristics

**Fig 1.**
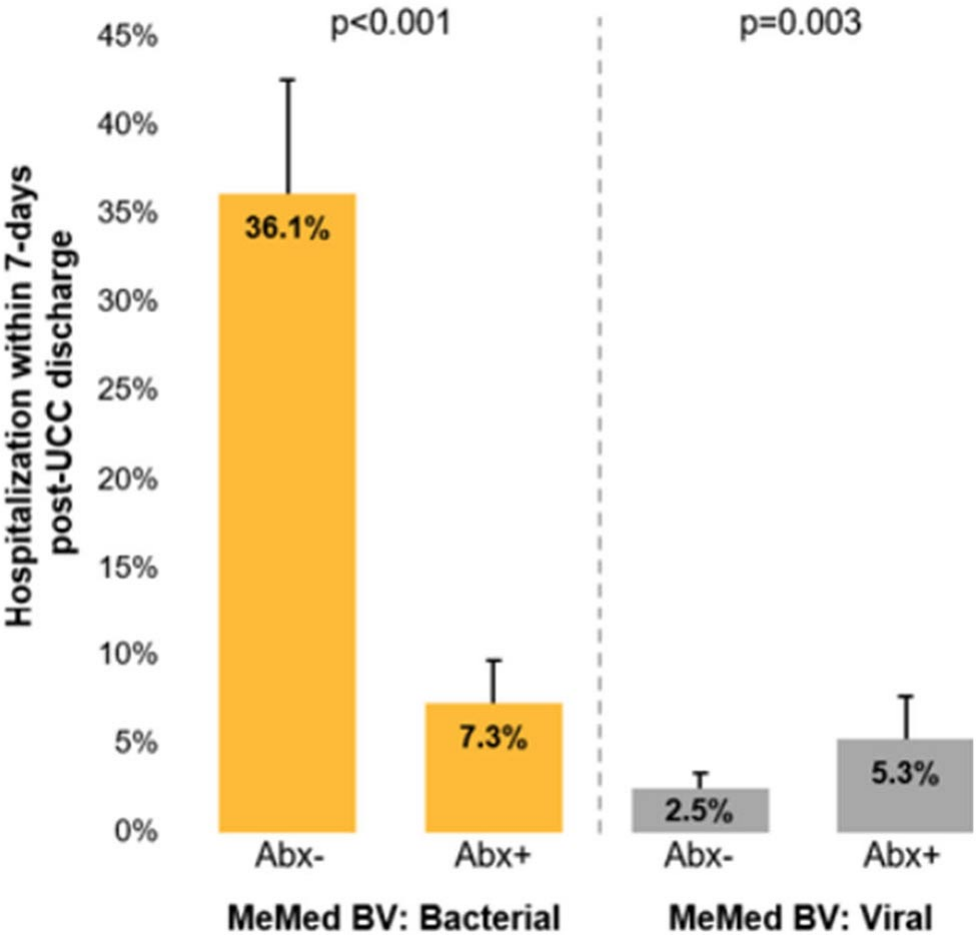
MeMed BV results, antibiotic prescription and hospitalization within 7-days.

**Methods:**

We retrospectively analyzed real-world data from adult patients who underwent MMBV testing during visits to 10 UCCs between April and December 2022. MMBV scores >65 indicate a bacterial infection or bacterial-viral co-infection; < 35 indicate viral infection; 35–65 are equivocal.

Concordance was defined as prescribing antibiotics at the UCC when the MMBV result indicated bacterial infection and not prescribing antibiotics when viral. Equivocal results were not included in the concordance calculation as physicians were trained to disregard this result in their decision making.

The primary outcome was hospitalization within 7-days post-UCC discharge.

**Results:**

A total of 3,758 patients received MMBV testing (59.3% female, median age 42 [IQR 31–58]); 858 (22.8%) had bacterial results, 2,404 (64.0%) viral, and 496 (13.2%) equivocal. Patients with bacterial MMBV results were older than those with viral results (median 51 vs. 39 years) and more often diagnosed with lower respiratory tract infections (LRTI) (29.6% vs. 9.4%; Table 1).

Among patients with bacterial MMBV results, antibiotic prescription was associated with significantly lower hospitalization rates within 7 days (7.3% vs. 36.1%, p< 0.001; Fig 1). Among those with viral MMBV results, not prescribing antibiotics was associated with a lower rate of LRTI diagnosis (4.2% vs. 29.5%) and with fewer hospitalizations (2.5% vs. 5.3%, p=0.003; Fig 1).

**Conclusion:**

Incorporation of MeMed BV into the decision-making process for managing UCC adult patients with acute infections is associated with improved patient care, reflected by fewer hospitalizations in post-UCC follow-up.

**Disclosures:**

All Authors: No reported disclosures

